# Loss of melanocortin receptor accessory protein 2 in melanocortin-4 receptor neurons protect from obesity-associated autonomic and cardiovascular dysfunctions

**DOI:** 10.1093/cvr/cvaf067

**Published:** 2025-04-17

**Authors:** Deng Fu Guo, Paul A Williams, Alexis Olson, Donald A Morgan, Hussein Herz, Jon Resch, Deniz Atasoy, Harald M Stauss, Julien A Sebag, Kamal Rahmouni

**Affiliations:** Department of Neuroscience and Pharmacology, University of Iowa Carver College of Medicine, 51 Newton Road, Iowa City, IA 52242, USA; Iowa City Veterans Affairs Health Care System, 51 Newton Road, Iowa City, IA 52242, USA; Department of Neuroscience and Pharmacology, University of Iowa Carver College of Medicine, 51 Newton Road, Iowa City, IA 52242, USA; Department of Neuroscience and Pharmacology, University of Iowa Carver College of Medicine, 51 Newton Road, Iowa City, IA 52242, USA; Department of Neuroscience and Pharmacology, University of Iowa Carver College of Medicine, 51 Newton Road, Iowa City, IA 52242, USA; Iowa City Veterans Affairs Health Care System, 51 Newton Road, Iowa City, IA 52242, USA; Department of Internal Medicine, University of Iowa Carver College of Medicine, 51 Newton Roand, Iowa City, IA 52242, USA; Department of Neuroscience and Pharmacology, University of Iowa Carver College of Medicine, 51 Newton Road, Iowa City, IA 52242, USA; Fraternal Order of Eagles Diabetes Research Center, University of Iowa Carver College of Medicine, 51 Newton Road, Iowa City, IA 52242, USA; Obesity Research and Educational Initiative, University of Iowa Carver College of Medicine, 51 Newton Road, Iowa City, IA 52242, USA; Iowa Neuroscience Institute, University of Iowa Carver College of Medicine, 51 Newton Road, Iowa City, IA 52242, USA; Department of Neuroscience and Pharmacology, University of Iowa Carver College of Medicine, 51 Newton Road, Iowa City, IA 52242, USA; Fraternal Order of Eagles Diabetes Research Center, University of Iowa Carver College of Medicine, 51 Newton Road, Iowa City, IA 52242, USA; Obesity Research and Educational Initiative, University of Iowa Carver College of Medicine, 51 Newton Road, Iowa City, IA 52242, USA; Iowa Neuroscience Institute, University of Iowa Carver College of Medicine, 51 Newton Road, Iowa City, IA 52242, USA; Department of Biomedical Sciences, Burrell College of Osteopathic Medicine, 3501 Arrowhead Dr., Las Cruces, NM, USA; Fraternal Order of Eagles Diabetes Research Center, University of Iowa Carver College of Medicine, 51 Newton Road, Iowa City, IA 52242, USA; Obesity Research and Educational Initiative, University of Iowa Carver College of Medicine, 51 Newton Road, Iowa City, IA 52242, USA; Iowa Neuroscience Institute, University of Iowa Carver College of Medicine, 51 Newton Road, Iowa City, IA 52242, USA; Department of Molecular Physiology and Biophysics, University of Iowa Carver College of Medicine, 51 Newton Road, Iowa City, IA, USA; Department of Neuroscience and Pharmacology, University of Iowa Carver College of Medicine, 51 Newton Road, Iowa City, IA 52242, USA; Iowa City Veterans Affairs Health Care System, 51 Newton Road, Iowa City, IA 52242, USA; Department of Internal Medicine, University of Iowa Carver College of Medicine, 51 Newton Roand, Iowa City, IA 52242, USA; Fraternal Order of Eagles Diabetes Research Center, University of Iowa Carver College of Medicine, 51 Newton Road, Iowa City, IA 52242, USA; Obesity Research and Educational Initiative, University of Iowa Carver College of Medicine, 51 Newton Road, Iowa City, IA 52242, USA; Iowa Neuroscience Institute, University of Iowa Carver College of Medicine, 51 Newton Road, Iowa City, IA 52242, USA

**Keywords:** Accessory proteins, Melanocortin receptors, Energy homeostasis, Insulin sensitivity, Sympathetic nervous system, Baroreflex sensitivity, Blood pressure, Heart rate

## Abstract

**Aims:**

The melanocortin receptor accessory protein 2 (MRAP2), which is abundantly expressed in the brain including the hypothalamus, has emerged as a key regulator of melanocortin-4 receptor (MC4R) activity. We sought to delineate the physiological significance of MRAP2 in MC4R neurons, with a particular focus on metabolic, autonomic and cardiovascular functions.

**Methods and results:**

Selective deletion of MRAP2 in MC4R neurons causes obesity that was associated with hyperphagia and impairment in glucose homeostasis and insulin sensitivity. MC4R agonist Melatonan II (MTII)-induced anorectic effects were blunted in mice lacking MRAP2 in MC4R neurons, whereas Celastrol retained its efficacy in reducing food intake and body weight. MRAP2 deletion also reduced baseline sympathetic nerve activity (SNA), particularly the SNA subserving the kidney. This was associated with reduced innervation of the kidney. In addition, MTII-induced increases in renal and brown adipose tissue (BAT) SNA as well as hepatic vagal nerve activity were significantly attenuated in MC4R neuron MRAP2-deficient mice. Transynaptic tracing revealed that MC4R neurons projecting to BAT and kidneys were localized to specific brain nuclei including the paraventricular nucleus of the hypothalamus, providing anatomical substrate for MRAP2 regulation of sympathetic outflow. Although the loss of MRAP2 in MC4R neurons did not affect arterial pressure, it caused a significant decrease in heart rate and baroreflex sensitivity. Finally, MRAP2 deficiency in MC4R neurons attenuated MTII-induced increase in arterial pressure and heart rate.

**Conclusion:**

These findings demonstrate that in addition to its role in energy balance and glucose homeostasis MRAP2 in MC4R neurons is crucial for cardiovascular autonomic regulation and is required for the development of obesity-associated hypertension and autonomic dysfunction.

## Graphical Abstract

**Graphical Abstract:**
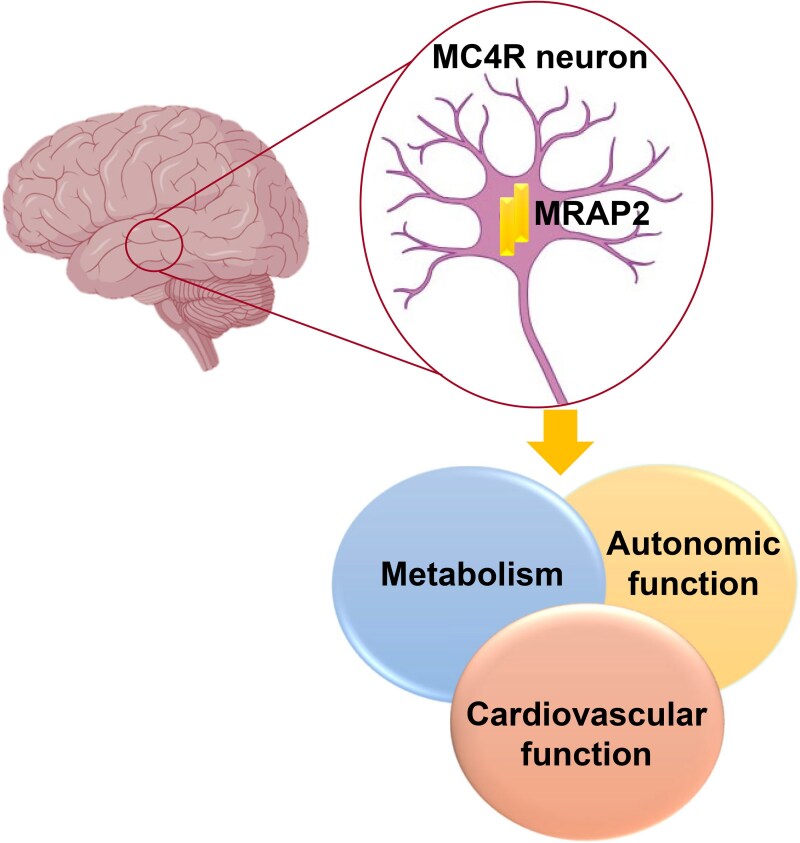

**Time of primary review: 65 days**

## 1. Introduction

According to the World Health Organization the worldwide prevalence of obesity has nearly tripled in the last 4–5 decades. In the USA, excess adiposity has increased steadily, and about two-thirds of the population is now considered overweight or obese.^1,2^ This represent a great public health concern because obesity is associated with comorbidities, especially type 2 diabetes and cardiovascular risks such as hypertension, a leading cause of death globally.^3,4^ This co-existence of metabolic and cardiovascular risks is a consequence of the tremendous influence that metabolic processes exert on the cardiovascular system.^5^ Estimates indicate that the continued trend in the prevalence of excess weight will exacerbate the burden of cardiovascular disease.^6,7^

Extensive evidence points to the central nervous system as a major player in the regulation of energy homeostasis. This involves complex neurocircuits that mediate the control of energy intake and expenditure. The melanocortin system is a key modulator of this neurocircuit. The hyperphagia and morbid obesity observed in humans and animals lacking key components of this system such as the melanocortin-4 receptor (MC4R) has reinforced the importance of the central melanocortin circuit for energy balance.^8,9^ Moreover, MC4R has emerged as a critical regulator of the autonomic nervous system and blood pressure.^10^ The activation of brain MC4R increases sympathetic nerve traffic and blood pressure, whereas genetic deletion or pharmacological blockade of MC4R prevents the rise in sympathetic traffic and blood pressure evoked by excess weight gain, highlighting the critical role of this receptor for obesity-induced sympathetic overdrive and hypertension^11–13^ Despite this, the exact processes underlying metabolic, autonomic, and cardiovascular regulation by MC4R are not well understood.

The melanocortin receptor accessory protein (MRAP) family, which is composed of two paralogs, MRAP1 and MRAP2, are single, transmembrane proteins that act as receptor molecular chaperones.^14,15^ In particular, MRAP2, which is abundantly expressed in the brain including the hypothalamus, has emerged as a key regulator of MC4R activity by influencing its trafficking and signalling.^16,17^ Consequently, the loss of MRAP2 function disrupts MC4R signalling, leading to obesity^18–20^ This obesity phenotype was recapitulated by *mrap2* gene deletion in various neuronal populations including SIM1 neurons and MC4R neurons.^19,21^ Conversely, overexpression of MRAP2 in MC4R neurons of the paraventricular nucleus of the hypothalamus (PVN) led to reduction in body weight due to a decrease in fat mass selectively in female mice.^22^ It should be noted, however, that unlike obese MC4R null mice, MRAP2 knockout mice displayed no change in food intake before the onset of obesity.^19,22^ Furthermore, while MC4R deficiency is associated with low/normal blood pressure and normal glucose, patients carrying deficient MRAP2 develop hypertension and hyperglycaemia.^20^ However, the contribution of MRAP2 in MC4R neurons to blood pressure regulation and the development of hypertension is unclear. Moreover, the role of MRAP2 in the control of autonomic function is not known.

Our current study was aimed at delineating the significance of MRAP2 in MC4R neurons, with a particular focus on autonomic, and cardiovascular functions. For this, we investigated the impact of MC4R neuron MRAP2 deletion on sympathetic nerve activity (SNA), and its downstream effects on blood pressure, heart rate, and baroreflex sensitivity. Parallel analysis of body weight and glucose homeostasis enabled a comprehensive assessment of the cardiometabolic consequences of MRAP2 deficiency in MC4R neurons.

## 2. Methods

### 2.1 Animals

All animal testing was performed in accordance with guidelines set by the National Institutes of Health and approved by The University of Iowa Animal Care and Use Committee. Mice were housed in groups of 2–5 per cage and maintained on a 12-h light–dark cycle with lights on at 6 am. Room temperature was maintained at 22°C. Food (normal chow) and water were available *ad libitum* except when the mice were fasted. *Mrap2* knockout (MRAP2^−/−^) mice and mice carrying floxed alleles of the *Mrap2* gene (MRAP2^fl/fl^) were generated by the Sanger Mouse Genetics Project as reported previously.^23^ ROSA (tdTomato fluorescence protein) reporter transgenic mice, which have a stop codon flanked by loxP sites preceding the start position of a tdTomato locus (Stop^fl/fl^ tdTomato), were obtained from the Jackson Laboratory. Cre recombination removes the stop site, leading to the expression of the red fluorescent td-Tomato protein.^24^ Mice expressing Cre recombinase driven by the *mc4r* promoter (MC4R^Cre^) were obtained from Dr Bradford Lowell.^25^

To obtain a selective *mrap2* gene knockout in MC4R neurons, female MRAP2^fl/fl^ mice were crossed with male MC4R^Cre^ mice. Male MC4R^Cre+^/MRAP2^wt/fl^ offspring were subsequently crossed with female MC4R^Cre−^/MRAP2^wt/fl^ mice to generate MC4R^Cre+^/MRAP2^fl/fl^ mice and control littermates (MC4R^Cre+^/MRAP2^wt/wt^, MC4R^Cre−^/MRAP2^wt/fl^, and MC4R^Cre−^/MRAP2^fl/fl^). These mice were further crossed to the tdTomato fluorescence protein reporter mice to visualize Cre recombinase activity and label MC4R neurons. Presence of the Cre transgene, floxed alleles of *mrap2* gene, and tdTomato was determined via PCR analysis of tail DNA under the following conditions: 94°C for 30 s, 60°C for 30 s, and 72°C for 45 s for 30 cycles. Primers used for genotyping are listed in the Supplementary material online, *Table S1*.

### 2.2 Intracerebroventricular (ICV) cannulation

Mice were anaesthetised using isoflurane (5% for induction; 1–2% to sustain). Once anaesthetised, each mouse was placed in a stereotaxic device and implanted with a stainless-steel cannula (25 gauge, 9 mm length) into the lateral brain ventricle (0.3 mm posterior and 1.0 mm lateral to bregma, and 3.0 mm below the surface of the skull) as described previously.^26,27^ Holes for the ICV cannula and screws were drilled in the skull using a handheld Dremel 7000 rotary tool. Screws were placed in the upper left and lower right quadrant of the skull to provide an anchor, and dental cement was applied to the skull up to the bump on the cannula to ensure it would not shift during recovery. Cannulae were obstructed using a 32G wire bent into an arch and secured in the dental cement. Mice were allowed to recover for at least 1 week prior to experimentation.

### 2.3 Body weight and food intake measurements

Growth curves were established by weighing mice once a week for 12 weeks starting at weaning (4 weeks of age). Body composition (fat mass and lean mass) was measured in conscious mice using nuclear magnetic resonance (LF50, Bruker Mini Spec).^28^ To measure food intake, mice were housed individually in their home cages. After 3 days of acclimatization to individual housing, daily food intake was measured over 4 days at 16 weeks of age. At the end of experiments, mice were sacrificed and various fat pads including gonadal, inguinal, peri-renal, and brown adipose tissue (BAT), and liver, were collected and weighed.

To assess the effects of Melatonan II (MTII, Milipore Sigma, cat. #M8693) and Celastrol (Cayman Chemical Company, cat. #: 34157-83-0) on food intake and body weight, mice were housed individually starting 1 week before baseline food intake, and body weight were measured daily for 5 consecutive days. One cohort of mice received ICV injections of MTII (2 μg/1 µL) or vehicle (1 µL) before the onset of the dark period. Food intake response to ICV MTII was measured 4 and 24 h after treatment, whereas body weight response was recorded after 24 h. Another cohort of mice received intraperitoneal (IP) injections of vehicle (1 μL/g body weight, twice per day for 4 days). One day after the end of vehicle treatment, IP injection of Celastrol (0.5 mg/kg body weight, twice daily for 4 days) was administered into the same mice. Food intake and body weight were recorded daily during the vehicle and Celastrol treatments.

### 2.4 Glucose and insulin tolerance tests

A glucose tolerance test was performed on overnight-fasted mice at 16 weeks of age. Blood samples were taken from the tail to measure blood glucose at baseline followed by the injection of D-glucose (Sigma Aldrich, 2 mg/kg body weight, IP). To obtain blood, a 1–2 mm piece of tissue was cut from the tail tip distal to the bone with sharp scissors. The tail was then gently massaged to produce blood (1–2 μL), which was collected directly on a glucose test strip (One Touch Ultra 1, LifeScan Inc.). For insulin tolerance test, mice were fasted for 5 h. Baseline glucose levels were measured before injection of insulin (Novo Nordisk, 0.5 U/kg body weight, IP). Blood glucose was measured at 15, 30, 60, and 120 min after injection of glucose or insulin.

### 2.5 Sympathetic and vagal nerve recordings

Mice anaesthetised with IP administration of Ketamine (91 mg/kg body weight) and Xylazine (9.1 mg/kg body weight) underwent intubation with PE-50 to provide an unimpeded airway for the mouse to spontaneously breathe O_2_-enriched room air. Next, a tapered micro-renathane tube (MRE-40, Braintree Scientific) was inserted into the right jugular vein for infusion of the sustaining anaesthetic agent: α-chloralose (initial dose: 12 mg/kg, then sustaining the dose of 6 mg/kg/h). Another tapered MRE-40 catheter, inserted into the left carotid artery and connected to a Powerlab via a pressure transducer, was used for continuous measurement of arterial pressure and heart rate. Core body temperature, monitored through a rectal probe, was maintained at 37.5°C.

Next, mice were equipped for direct multifiber SNA recording from the nerves subserving BAT or the kidney. The nerve fascicles that innervate the interscapular BAT was accessed through an incision in the nape of the neck. In other mice, the post-ganglionic nerve fibres that subserve the left kidney (renal SNA) was accessed through a retro-peritoneal incision. In another cohort of mice, hepatic vagal nerve activity was recorded as described previously.^27,29^ A midline incision was made from the mid-abdomen to the xyphoid process. The stomach was exteriorized, and the liver lobes retracted to reveal the oesophagus extending from the diaphragm to the stomach fundus. On the ventral surface of the oesophagus, the hepatic vagal nerve was carefully isolated, placed on a bipolar 36-gauge platinum–iridium electrodes and finally sealed with a silicone gel as the vagal nerve was branching to the nearest liver lobe.

Each sympathetic or vagal nerve fascicle was carefully isolated from surrounding connective tissues. A bipolar platinum–iridium electrode (36-gauge platinum–iridium, A-M Systems) was then suspended under the nerve and secured with silicone gel (Kwik-Cast, WPI). The electrodes were attached to a high-impedance probe (HIP-511, Grass Instruments) and the nerve signal was amplified 10^5^ times and filtered at a 100 and 1000 Hz cut-off with a Grass P5 AC pre-amplifier. The amplified and filtered nerve signal was routed to a speaker system and to an oscilloscope (model 54501A, Hewlett-Packard) to monitor the audio and visual quality of the nerve recordings and for quantification purposes. The amplified, filtered nerve signal was also directed to a MacLab analogue-digital converter (Model 8S, AD Instruments) containing the software (MacLab Chart Pro; Version 7.0) that utilizes a cursor to analyse the number of spikes/second that exceeds the background noise threshold. Baseline BAT and renal SNA as well as hepatic vagal nerve activity were recorded over a 30-min control period before treatments with ICV MTII (2 μg) or vehicle (2 μL). After treatments, SNA and vagal nerve activity were recorded continuously and quantified at 15 min intervals over 4 h.

Other cohorts of mice were used to measure baroreceptor reflex sensitivity as reported before.^30^ After baseline renal SNA was obtained, mice were treated intravenously with vehicle (saline, 10 μL) followed by escalating doses of sodium nitroprusside (SNP: 0.01, 0.05, 0.1, and 0.2 μg) and phenylephrine (PE: 0.01, 0.05, 0.1, and 0.2 μg). Time was allowed between injections (vehicle, SNP, and PE) for the peak effect on SNA, blood pressure, and heart rate to return to baseline values before the next injection was given. The quantitative measure of baroreflex sensitivity was provided by the slope of the fitted line, which was expressed as the percent change in renal SNA (spikes/sec) or heart rate (bpm) per mmHg change in mean arterial blood pressure induced by vehicle, SNP, and PE. The fitted line was made using Microsoft Excel. At the end of the experiment, mice were sacrificed using an overdose of the chloralose solution (270 mg/kg, intravenously). Any residual nerve activity after death was considered as background noise and subtracted from the measured SNA.

### 2.6 Radiotelemetry blood pressure recording

A radiotelemetry transmitter (PA-C10, Data Science Instruments) was used to directly measure arterial pressure, heart rate, and locomotor activity as previously described.^31^ Mice were anaesthetised with 5% isoflurane and maintained on 1–2% isoflurane until the completion of the implant surgery. The left carotid artery was isolated and the catheter’s tip was carefully inserted through the blood vessel for a distance of ∼9 mm until the blunt, open end of the catheter was positioned where the left carotid artery immediately branches from the aortic arch. After achieving the proper placement of the catheter and hearing a strong pulsatile radio signal (when the unit was turned on), the telemetry catheter was finally secured in place with 4-0 silk sutures. The transmitter unit was tunnelled sub-dermally from the neck incision down to the right flank of the abdominal region. The neck incision was closed with a 4-0 polyglycolic acid absorbable suture and further sealed with tissue adhesive (Vet-bond). Mice were allowed to recover in single unit cages for at least 10 days.

Arterial pressure, heart rate, and locomotor activity were recorded continuously for 2–3 days in conscious unrestrained conditions. To evaluate autonomic nervous system activity and baroreflex sensitivity, blood pressure, and heart rate were recorded continuously for 1 h at higher frequency (2000 Hz). To assess the effect of MC4R activation on arterial pressure and heart rate, mice were treated with ICV MTII (2 μg) or vehicle (2 μL).

### 2.7 Tracing experiments

To identify co-localization between organ-associated MC4R neurons, pseudorabies virus (PRV) expressing a green-fluorescent reporter protein (GFP) [PRV-152, referred to as PRV–GFP, Centre for Neuroanatomy and Neurotropic Viruses (CNNV), http://www.cnnv.pitt.edu/faqs.htm, titer = 2.69 × 10^9^ pfu/mL] was injected into either the kidneys or BAT of MC4R^Cre^/tdTomato reporter mice, under isoflurane anaesthesia (5% for induction; 1–2% to sustain). The kidneys were exposed via a retro-peritoneal incision, and for the BAT an incision was made posterior to the interscapular fat pad. Once the organ was exposed a Hamilton syringe (80135, Hamilton Company Inc., Reno, Nevada) was used for the inoculation. For the kidneys, 0.5 μL (two injections of 0.25 μL) of PRV solution was injected into the cortex of the left and right kidney, while 0.25 μL was injected into both the left and right BAT. Sutures were then used to close all incisions. Post-operatively the animals were maintained in a BSL2 facility and checked each post-operative day. Observations of symptoms and body weight were utilized to assess each animal and determine the endpoint (sometime between post-operative Days 4 and 7). Once an animal reached its endpoint, it was euthanized using ketamine (250–300 mg/kg)/Xylazine (25–30 mg/kg) cocktail, IP, and perfused with cold phosphate-buffered saline (PBS), followed by 4% paraformaldehyde (PFA). After extraction, the brains were maintained in 4% PFA overnight at 4°C and then incubated in a 30% sucrose for 3 days at 4°C. Brain specimens were sectioned at 50 μm on a vibratome (Leica VT 1000S). Sections were then stained for GFP using a rabbit anti-GFP primary antibody (Invitrogen, Carlsbad, California, A6455, 1:250 dilution) and a goat anti-rabbit secondary antibody conjugated with Alexaflora 488 (Invitrogen, A11034, 1:500 dilution). All antibodies were diluted in a blocking buffer (95% PBS and 5% goat serum with 50 μL Triton X-100 added). Sections were mounted on slides and a standard mounting media (Vector Laboratories, Newark, California, Vectashield Plus with 4′,6-diamidino-2-phenylindole) applied prior to cover-slipping. A slide scanner (Olympus VS200) was used to obtain overall images of tdTomato and GFP. Detailed imaging of nuclei was obtained using a confocal microscope (Zeiss 880). Contract and brightness of the images were adjusted using ImageJ.

### 2.8 Tissue clearing technique

Mice are perfused with 4% PFA, and extracted kidneys and BAT were further fixed with 4% PFA overnight followed by washing with PBS for 1 h twice and then with 20, 40, 60, and 80% methanol (in PBS) for 1 h and finally with 100% methanol for 1 h twice. Samples were then bleached with 5% H_2_O_2_ in 20% dimethyl sulfoxide (DMSO)/methanol (1 vol 30% H_2_O_2_/1 vol DMSO/4 vol methanol, ice cold) at 4°C overnight. After bleaching, samples were washed in methanol for 1 h twice, in 20% DMSO/methanol for 1 h twice, then in 80, 60, 40 and 20% methanol (in PBS) for 1 h twice. Next, samples were incubated with tyrosine hydrolase (TH) antibody (Millpore Corp., Burlington, Massachusetts, cat.#: AB152, 1: 20 dilution) in blocking solution, (PBS/0.2% Triton X-100, 6% of donkey serum and 1% DMSO) for 7 days at 37°C. Samples were washed with blocking solution four times for 1 h, then overnight, and inculcated with second antibody Alexa Fluor 647 donkey anti-rabbit IgG (Life Technologies Corporation, Carlsbad, California, cat.#: A31573, 1: 125 dilution) for 6 days at 37°C. Samples were next washed with blocking solution four times for 1 h, then overnight, and dehydrated 20, 40, 60, and 80% methanol (in PBS) for 1 h, 100% methanol for 1 h twice, 66% DiChloroMethane (DCM)/34% methanol for 3 h, 100% DCM 15 min twice, and finally with DiBenzylEther. TH staining was acquired with Light-sheet microscopy (LaVison). TH volume and number of voxels were analysed with Imaris software.

### 2.9 Statistical analysis

Results are expressed as mean ± standard error of the mean. Normal distribution of data was assessed before statistical analysis. Data were analysed using *t*-test and 1- or 2-way analysis of variance (ANOVA) with or without repeated measures on GraphPad PRISM 9.1.0. When ANOVA reached significance, a *post hoc* comparison was made using the Fisher test or Tukey’s test. A *P*-value < 0.05 was considered to be statistically significant.

## 3. Results

### 3.1 MRAP2 deletion in MC4R-positive neurons induces obesity

In order to evaluate the role of MRAP2 in MC4R-containing neurons, we generated mice lacking the *mrap2* gene in MC4R-positive neurons (MC4R^Cre^/MRAP2^fl/fl^). This was achieved by crossing the MRAP2^fl/fl^ mice with mice expressing Cre recombinase specifically in MC4R neurons (MC4R^Cre^). We first visualized Cre-mediated recombination by crossing tdTomato reporter mice with MC4R^Cre^ mice. The presence of MC4R^Cre^ causes Cre recombination that inactivate the *mrap2* gene while removing the STOP coding preceding the tdTomato locus in MC4R-containing neurons. Analysis of fluorescent tdTomato driven by MC4R^Cre^ confirmed its presence in brain nuclei that are known to express the MC4R including the hypothalamus, thalamus, cortex, hippocampus, mid-brain, and brainstem regions (see Supplementary material online, *Figure S1* and *Table S2*).

**Figure 1:**
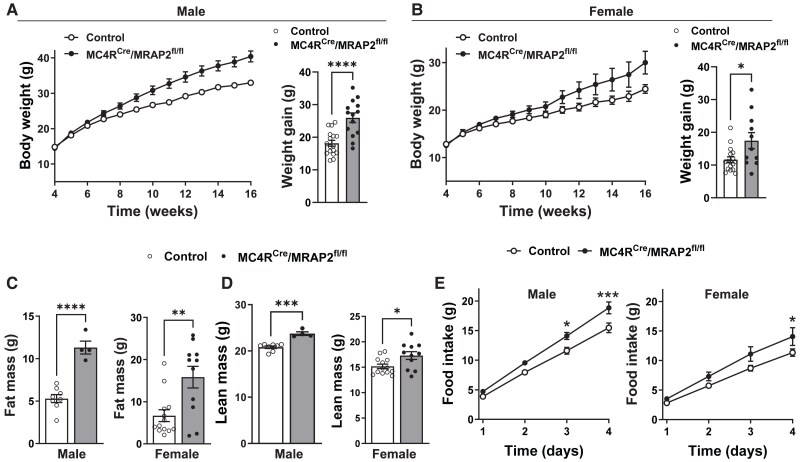
Loss of MRAP2 in MC4R neurons causes obesity. (*A* and *B*) growth curve and weight gain (at 16 weeks relative to 4 weeks of age) of male (*A*) and female (*B*) mice. (*C* and *D*) Fat mass (*C*) and lean mass (*D*) of male and female mice. (*E*) Cumulative daily food intake of male (*n* = 11 control and 7 MC4R^Cre^/MRAP2^fl/fl^) and female (*n* = 10 control and 6 MC4R^Cre^/MRAP2^fl/fl^) mice. Two-way ANOVA with repeated measure or Student’s *t*-test was used for statistical analysis. *P < 0.05, **P < 0.01, ***P < 0.001, and ****P < 0.0001.

To investigate the physiological consequences of specific MC4R MRAP2 ablation on energy homeostasis, we measured body weight of MC4R^Cre^/MRAP2^fl/fl^ mice and littermate controls beginning at weaning. There was no difference in body weight between MC4R^Cre^/MRAP2^fl/fl^ mice and controls (males: 14.4 ± 0.6 and 14.8 ± 0.4 g, respectively, *P* = 0.28; females: 12.5 ± 0.4 and 12.8 ± 0.4 g, respectively, *P* = 0.31). However, around 6 weeks of age body weight begin to diverge, with MC4R^Cre^/MRAP2^fl/fl^ mice gaining more weight compared to their respective controls (*Figure 1A* and *B*). This divergence in body weight continued over the 16-week course of the study, leading to both male and female MC4R^Cre^/MRAP2^fl/fl^ mice gaining significantly more weight than their littermate controls.

Consistent with the increased body weight, fat mass was significantly higher in male and female MC4R^Cre^/MRAP2^fl/fl^ mice (*Figure 1C*). Fat pads weight confirmed the increase in adiposity in male and female MC4R^Cre^/MRAP2^fl/fl^ mice (see Supplementary material online, *Figure S2A* and *B*). Notably, MC4R^Cre^/MRAP2^fl/fl^ mice displayed increased lean mass (*Figure 1D*). In addition, MC4R^Cre^/MRAP2^fl/fl^ mice exhibited elevated liver weight, but this was statistically significant in males only (see Supplementary material online, *Figure S2A* and *B*). Both male and female MC4R^Cre^/MRAP2^fl/fl^ mice were hyperphagic as indicated by the increased food intake (*Figure 1E*). These data demonstrate that MRAP2 in MC4R neurons is required for energy homeostasis.

### 3.2 Loss of MRAP2 in MC4R neurons alter insulin sensitivity and glucose homeostasis

**Figure 2:**
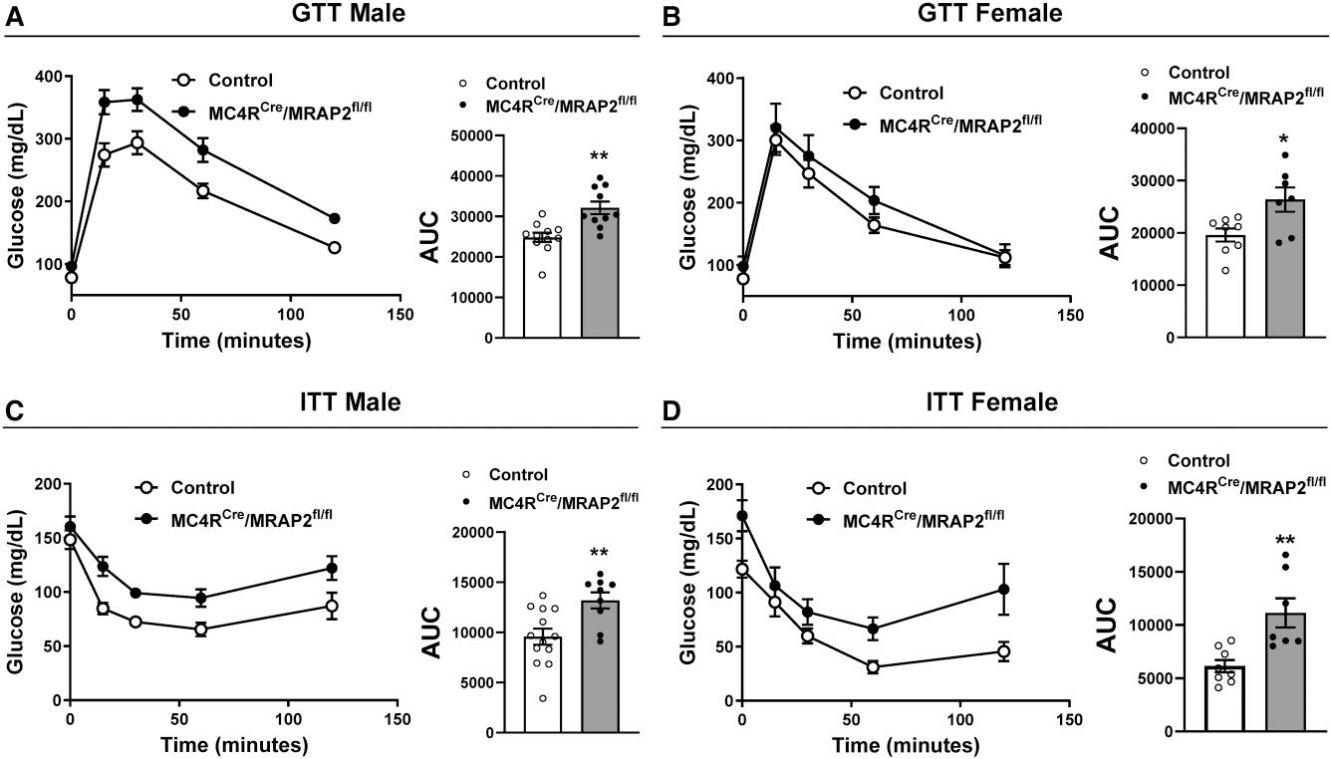
The MC4R neuron MRAP2 is necessary for glucose homeostasis. (*A* and *B*) Glucose tolerance test (GTT) of male (*A*) and female (*B*) mice. (*C* and *D*) Insulin tolerance test (ITT) of male (*C*) and female (*D*) mice. Two-way ANOVA with repeated measure or Student’s *t*-test was used for statistical analysis. *P < 0.05 and **P < 0.01.

Next, we investigated the effect MRAP2 deletion in MC4R neurons on glucose homeostasis. Fasting blood glucose was elevated in MC4R^Cre^/MRAP2^fl/fl^ mice, reaching statistical significance in males (96 ± 5 vs. 78 ± 4 mg/dL, *P* = 0.01) but not females (98 ± 17 vs. 78 ± 5 mg/dL, *P* = 0.11). The glucose tolerance test revealed that male MC4R^Cre^/MRAP2^fl/fl^ mice were glucose intolerant as indicated by the diminished glucose clearance following IP glucose injection (*Figure 2A*). This was further confirmed by the calculation of the area under the curve. Female MC4R^Cre^/MRAP2^fl/fl^ mice exhibited less marked glucose intolerance (*Figure 2B*). We next performed an insulin tolerance test which showed that both male and female MC4R^Cre^/MRAP2^fl/fl^ mice were insulin-resistant as indicated by the attenuated decrease in blood glucose following insulin injection (*Figure 2C* and *D*). These findings indicate that MRAP2 in MC4R neurons is necessary for glucose homeostasis and insulin sensitivity.

### 3.3 MC4R neuron MRAP2 deficiency affects the anorectic effect of MTII, but not that of Celastrol

**Figure 3:**
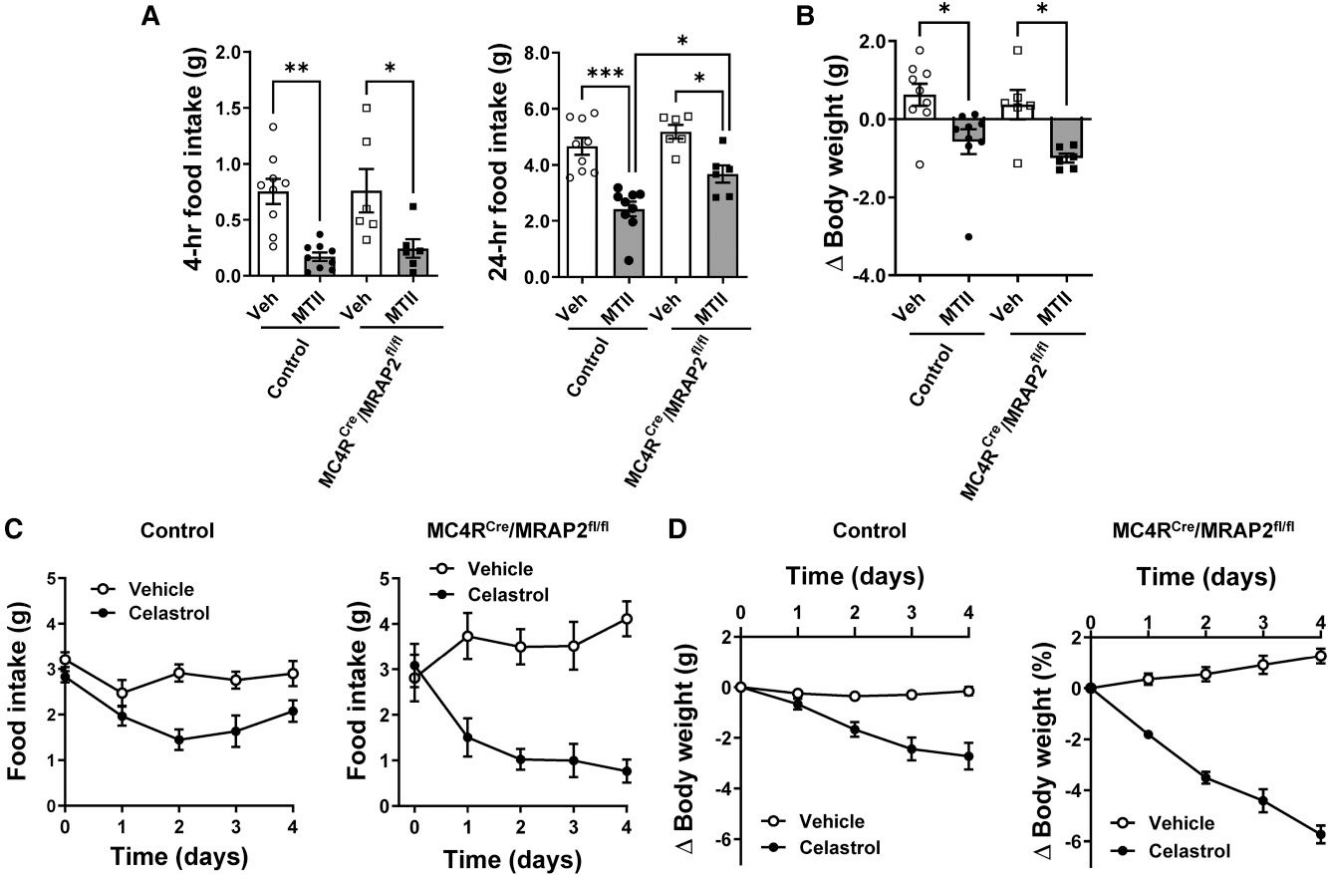
MC4R neuron MRAP2 deficiency affects specifically the anorectic of MC4R stimulation. (*A* and *B*) Effect of MTII (ICV, 2 µg) vs. vehicle on 4 and 24 h food intake (*A*) and body weight (*B*) of control and MC4R^Cre^/MRAP2^fl/fl^ male mice. (*C* and *D*) Effect of Celastrol (IP, 0.5 mg/kg body weight) vs. vehicle treatment on daily food intake (*C*) and body weight (*D*) of control (*n* = 7) and MC4R^Cre^/MRAP2^fl/fl^ (*n* = 6) male mice. One- or Two-way ANOVA with repeated measure was used for statistical analysis. *P < 0.05, **P < 0.01, and ***P < 0.001.

Given the previously described role of MRAP2 in regulating MC4R function,^15,23^ we assessed whether the loss of MRAP2 interferes with the physiological responses evoked by the stimulation of the MC4R. We first measured the ability of the MC4R agonist, MTII, to decrease food intake and body weight. ICV injection of MTII caused a significant decrease in food intake, measured at 4 and 24 h after treatment (*Figure 3A*). Interestingly, ICV MTII-induced decrease in food intake was blunted in the MC4R^Cre^/MRAP2^fl/fl^ mice at 24 h, but not at 4 h. As expected, ICV MTII also caused a significant decrease in body weight. However, there was no difference between MC4R^Cre^/MRAP2^fl/fl^ mice and controls (*Figure 3B*).

We previously demonstrated that Celastrol, a small molecule found in the roots of the *Tripterygium wilfordii* plant, evokes a significant weight loss in mice with acquired and genetic forms of obesity including MC4R-deficient mice.^32^ Thus, we tested whether Celastrol decreases food intake and body weight in MC4R^Cre^/MRAP2^fl/fl^ mice. Compared with vehicle, Celastrol treatment for 4 days induced a significant decrease in food intake and body weight in MC4R^Cre^/MRAP2^fl/fl^ mice (*Figure 3C* and *D*). Interestingly, the anorectic and weight-reducing effects of Celastrol seem more pronounced in MC4R^Cre^/MRAP2^fl/fl^ mice relative to controls, but when body weight changes were expressed relative to baseline the weight loss was statistically different only on Day 4 (see Supplementary material online, *Figure S4*). These data show the efficacy of Celastrol to cause weight loss in mice lacking MRAP2 in the MC4R neurons. These findings also indicate that the attenuated anorectic effect of MTII is specific.

### 3.4 Sympathetic and parasympathetic effects of MRAP2 deletion in MC4R neurons

**Figure 4:**
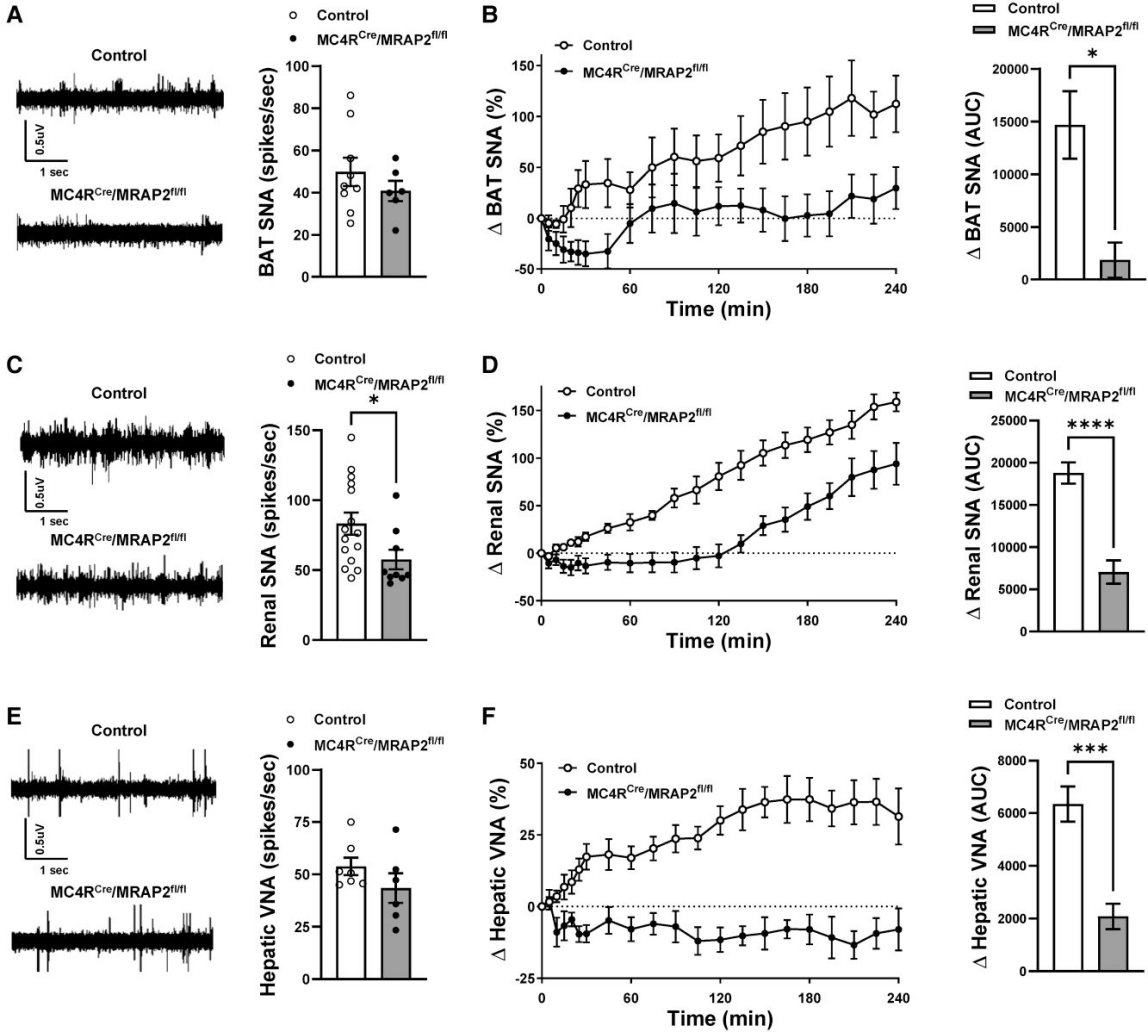
MC4R neuron MRAP2 deficiency attenuates the sympathetic responses to MC4R stimulation. (*A*) Representative neurograms and quantification of baseline BAT SNA of control and MC4R^Cre^/MRAP2^fl/fl^ male mice. (*B*) Effect of MTII (ICV, 2 µg) vs. vehicle on BAT SNA of control (*n* = 8) and MC4R^Cre^/MRAP2^fl/fl^ (*n* = 5) male mice. (*C*) Representative neurograms and quantification of baseline renal SNA of control and MC4R^Cre^/MRAP2^fl/fl^ male mice. (*D*) Effect of MTII (ICV, 2 µg) vs. vehicle on renal SNA of control (*n* = 7) and MC4R^Cre^/MRAP2^fl/fl^ (*n* = 7) male mice. (*E*) Representative neurograms and quantification of baseline hepatic vagal nerve activity (VNA) of control and MC4R^Cre^/MRAP2^fl/fl^ male mice. (*F*) Effect of MTII (ICV, 2 µg) vs. vehicle on hepatic VNA of control (*n* = 7, 3 males and 4 females) and MC4R^Cre^/MRAP2^fl/fl^ (*n* = 7, 3 males and 4 females) mice. Two-way ANOVA with repeated measure or Student’s *t*-test was used for statistical analysis. *P < 0.05, ***P < 0.001, and ****P < 0.0001.

Next, we examined the consequence of MRAP2 deletion in MC4R neurons on the control of SNA. Baseline SNA subserving thermogenic BAT trended to be lower in MC4R^Cre^/MRAP2^fl/fl^ mice relative to controls (*P* = 0.17, *Figure 4A*). ICV injection of MTII increased BAT SNA in control mice, but this response was nearly absent in the MC4R^Cre^/MRAP2^fl/fl^ mice (*Figure 4B*). Similarly, global MRAP2 knockout mice displayed significantly attenuated BAT SNA response to ICV MTII (see Supplementary material online, *Figure S3*). To determine whether this attenuated response to MC4R activation was specific to BAT SNA, we examined renal SNA at baseline and in response to MTII. Interestingly, baseline renal SNA was significantly lower in MC4R^Cre^/MRAP2^fl/fl^ mice relative to controls (*Figure 4C*). Moreover, ICV injection of MTII resulted in a slow but robust increase in renal SNA in control mice (*Figure 4D*). MC4R^Cre^/MRAP2^fl/fl^ mice display a bunted MTII-induced increase in renal SNA. Thus, absence of MRAP2 interferes with the BAT and renal sympathetic responses induced by stimulation of the brain MC4R.

To test whether the effects of MRAP2 deletion in MC4R neurons are specific to the sympathetic nervous system, we analysed the parasympathetic nervous system of MC4R^Cre^/MRAP2^fl/fl^ mice. No significant difference was observed in baseline hepatic vagal nerve activity between MC4R^Cre^/MRAP2^fl/fl^ mice and controls (*Figure 4E*). However, the increase in hepatic vagal activity evoked by MTII was significantly blunted in MC4R^Cre^/MRAP2^fl/fl^ mice relative to controls (*Figure 4F*). These findings indicate that MRAP2 is also required for proper control of the parasympathetic nervous system by MC4R neurons.

**Figure 5:**
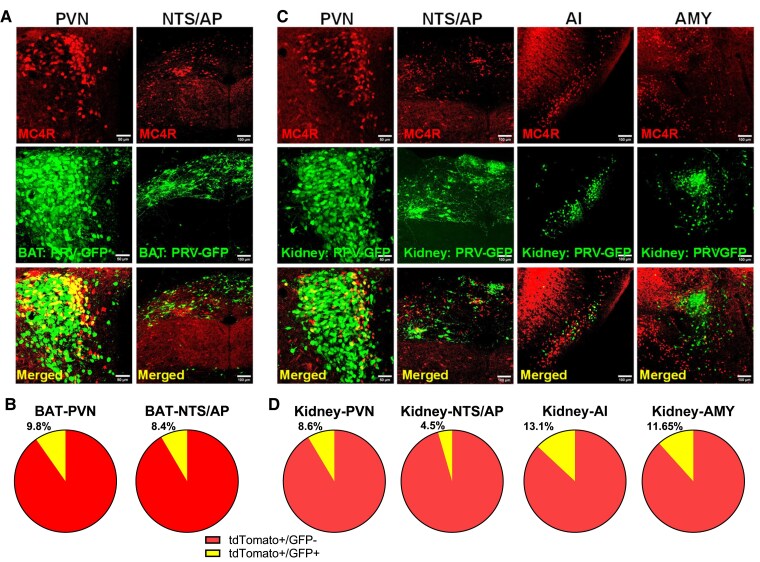
Identification of MC4R neurons in the central autonomic circuit. (*A* and *B*) Representative images (*A*) and quantification (*B*) of BAT associated MC4R neurons of the PVN and nucleus tractus solitarius (NTS)/AP in MC4R^Cre^/tdTomato reporter mice. *n* = 6 (3 males, 3 females). (*C* and *D*) Representative images (*C*) and quantification (*D*) of kidney associated MC4R neurons of PVN, NTS/AP, agranular insular cortex (AI) and amygdala (AMY) in MC4R^Cre^/tdTomato mice. *n* = 7 (4 males, 3 females). Scale bars: 50 μm. Contract and brightness of the images were adjusted on ImageJ.

We then took advantage of our mouse model that has the MC4R neurons labelled with tdTomato to explore the anatomical basis for the control by these neurons of BAT and renal SNA using transsynaptic tracing coupled with immunohistochemical labelling. Injection of attenuated pseudorabies expressing GFP (PRV–GFP) into BAT or the kidneys of MC4R^Cre^/tdTomato reporter mice revealed a specific subset of dual-labelled MC4R-tdTomato/PRV–GFP neurons in restricted number of nuclei at various levels of the brain (*Figure 5*). Interestingly, ∼10% of MC4R neurons co-expressed PRV–GFP (after BAT or kidney injection) in the PVN and tractus solitarius/area postrema (AP) (*Figure 5A* and *B* and Supplementary material online, *Figure S5A*). Intriguingly, after injection into the kidneys, but not BAT, PRV–GFP/MC4R co-localization (5–10% of MC4R neurons) was also detected in the agranular insular cortex and the amygdala (*Figure 5C* and *D* and Supplementary material online, *Figure S5B*). No co-localization was detected in any of the many other nuclei that contain MC4R neurons following the inoculation of BAT or kidneys with PRV–GFP. Therefore, although MC4R neurons are widely distributed throughout the brain, those projecting to BAT and kidney appear to be localized to only a few specific nuclei. This suggests that MRAP2 may regulate sympathetic outflow subserving these organs through a small number of distinct MC4R neurons that are part of the central autonomic network.

**Figure 6:**
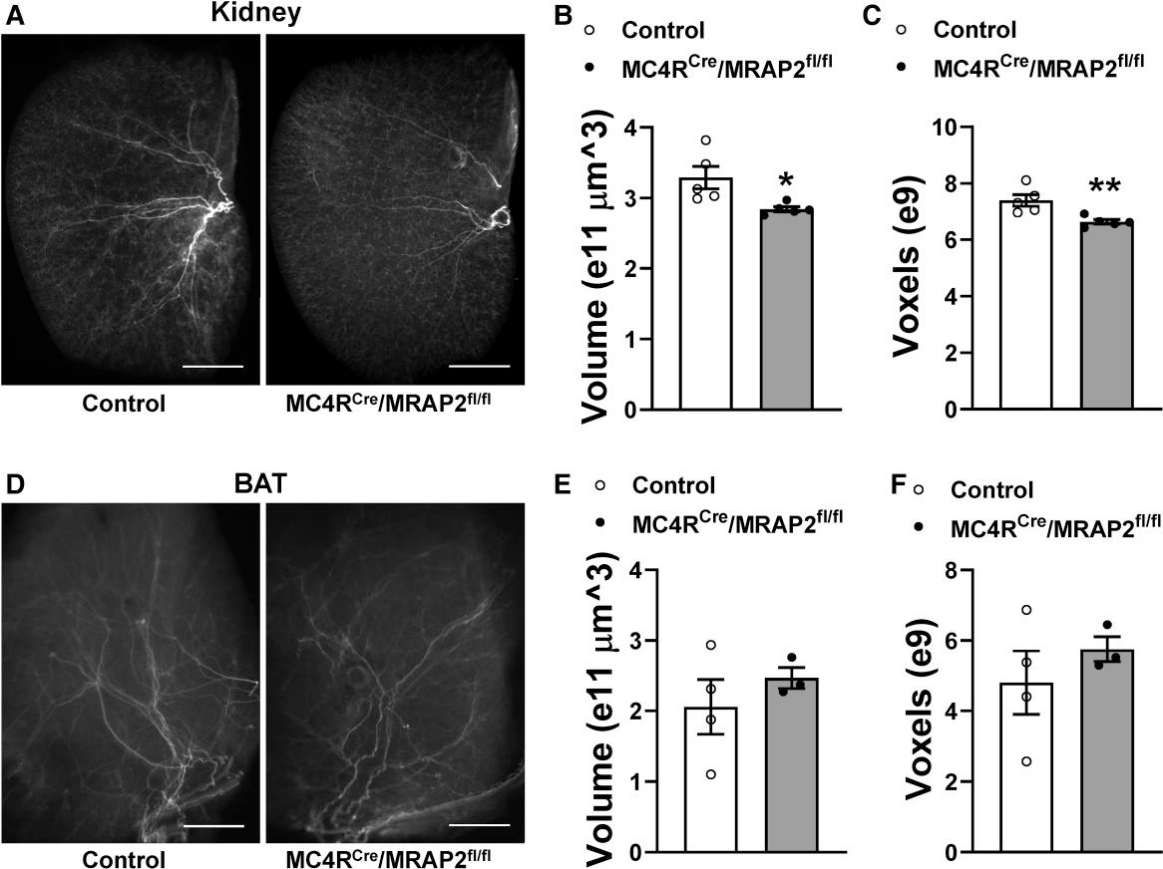
Loss of MC4R neuron MRAP2 reduce sympathetic innervation of the kidney. (*A–C*) Representative images (*A*) and quantification (*B* and *C*) of TH immunostaining in the kidney of control and MC4R^Cre^/MRAP2^fl/fl^ mice (*n* = 5 mice/group). (*D–F*) Representative images (*D*) and quantification (*E* and *F*) of TH immunostaining in the BAT of control and MC4R^Cre^/MRAP2^fl/fl^ mice (*n* = 3 MC4R^Cre^/MRAP2^fl/fl^ mice and 4 control mice). Scale bars: 1.5 mm. Student’s *t*-test was used for statistical analysis. *P < 0.05 and **P < 0.01.

We next imaged sympathetic innervation of BAT and kidney by pairing whole-mount tissue clearing with light-sheet microscopy.^33^ Immunolabelling for TH was used to mark sympathetic neurons. These studies revealed a significant decrease in sympathetic innervation of the kidney in MC4R^Cre^/MRAP2^fl/fl^ mice relative to controls (*Figure 6A–C*). In contrast, no difference was observed in sympathetic BAT innervation between MC4R^Cre^/MRAP2^fl/fl^ and control mice (*Figure 6D–F*).

### 3.5 Haemodynamic and autonomic consequences of MC4R neuron MRAP2 deficiency

**Figure 7:**
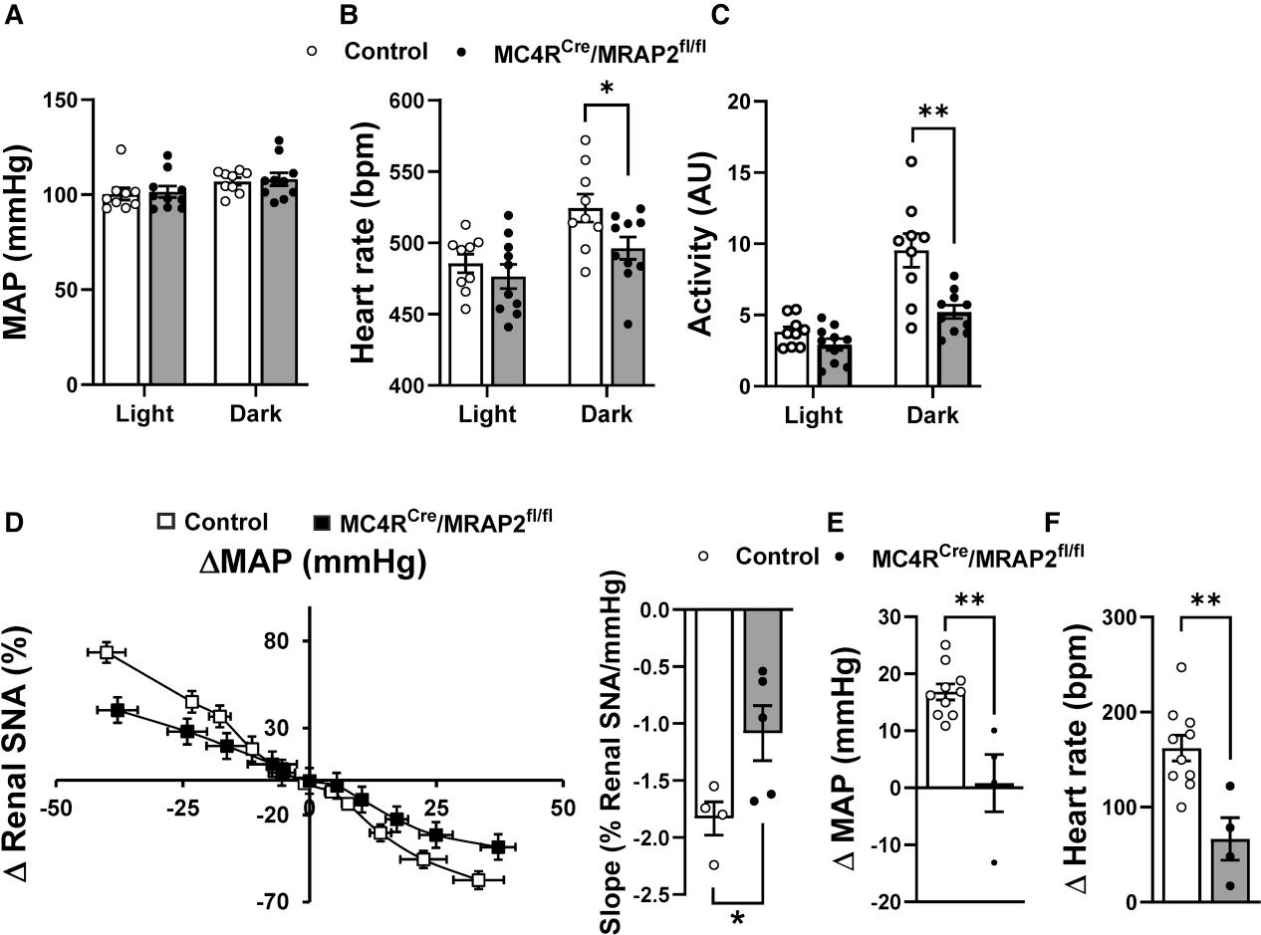
MC4R neuron MRAP2 deficiency protects from hypertension and pressor effect of MC4R stimulation. (*A–C*) Baseline telemetry mean arterial pressure (MAP, *A*), heart rate (*B*), and physical activity (*C*) of control and MC4R^Cre^/MRAP2^fl/fl^ male mice (*n* = 10 MC4R^Cre^/MRAP2^fl/fl^ mice and 9 controls). (*D*) Baroreceptor reflex sensitivity based on the change in renal SNA evoked by the increase and decrease in MAP induced by phenylephrine and sodium nitroprusside, respectively, and related slope of control and MC4R^Cre^/MRAP2^fl/fl^ male mice (*n* = 5 MC4R^Cre^/MRAP2^fl/fl^ mice and 4 controls). (*E* and *F*) Effects of MTII (ICV, 2 µg) vs. vehicle on MAP (*E*) and heart rate (*F*) of control and MC4R^Cre^/MRAP2^fl/fl^ male mice (*n* = 4 MC4R^Cre^/MRAP2^fl/fl^ mice and 10 controls). Two-way ANOVA with repeated measure or Student’s *t*-test was used for statistical analysis. *P < 0.05 and **P < 0.01.

The observation of reduced renal SNA in MC4R^Cre^/MRAP2^fl/fl^ mice prompted us to measure their blood pressure and heart rate. However, mean arterial pressure was similar between MC4R^Cre^/MRAP2^fl/fl^ mice and controls during the light and dark phases (*Figure 7A*). There was also no difference in systolic and diastolic arterial pressure (see Supplementary material online, *Figure S6A* and *B*). On the other hand, heart rate was significantly lower in MC4R^Cre^/MRAP2^fl/fl^ mice during the dark phase only (*Figure 7B*). MC4R^Cre^/MRAP2^fl/fl^ mice also displayed significant reduction in their locomotor activity during the dark phase (*Figure 7C*).

Power spectral analysis revealed a trend towards decreased low to high frequency (LF/HF) ratio of heart rate variability in MC4R^Cre^/MRAP2^fl/fl^ mice (see Supplementary material online, *Figure S7A*). Because there was no difference in LF spectral power (175 ± 78 vs. 110 ± 23 ms^2^, *P* = 0.41) but a trend towards higher HF spectral power (108 ± 45 vs. 25 ± 7 ms^2^, *P* = 0.10), the decreased LF/HF ratio indicates higher cardiac parasympathetic tone. Using the sequence technique—which utilizes spontaneous, physiological fluctuations of blood pressure, and cardiac interval—the ratio of systolic blood pressure fluctuations during non-baroreflex sequences to systolic blood pressure fluctuations during baroreflex sequences were calculated. This ratio—the baroreceptor-blood pressure (AP) reflex index—is <1 when blood pressure fluctuations during baroreflex sequences are larger than those during non-baroreflex sequences, indicating impaired baroreflex function. The significance of the baroreceptor-BP reflex index over the baroreceptor-heart rate reflex sensitivity is that it measures the true effect of the baroreceptor reflex on buffering arterial pressure fluctuations—which is the physiologic function of the baroreceptor reflex. This index showed a trend (*P* = 0.08) towards decrease in MC4R^Cre^/MRAP2^fl/fl^ mice relative to controls (see Supplementary material online, *Figure S7B*), indicating that the ability of the baroreceptor reflex to buffer blood pressure fluctuations is impaired. To further assess baroreceptor reflex function, we also measured the responses of renal SNA and heart rate to changes in arterial pressure evoked by infusion of SNP and PE, and these confirmed the decreased baroreceptor-renal SNA and baroreceptor-heart rate reflex sensitivity in MC4R^Cre^/MRAP2^fl/fl^ mice (*Figure 7D* and Supplementary material online, *Figure S7C*). Collectively, these data indicate that loss of the MC4R neuron MRAP2 affects activity of the autonomic nervous system and attenuates baroreflex sensitivity.

Finally, we tested whether the loss of MRAP2 in MC4R neurons affects the increase in blood pressure and heart rate induced by MCR4R activation. In control mice, ICV injection of MTII caused a robust increase in arterial pressure and heart rate as expected (*Figure 7E* and *F*). Remarkably, these responses were significantly blunted in MC4R^Cre^/MRAP2^fl/fl^ mice. This shows that MC4R neuron MRAP2 is required for the pressor and tachycardic responses evoked by MC4R stimulation.

## 4. Discussion

The most important new finding in the current study is the observation that loss of the MC4R neuron MRAP2 protects mice from obesity-associated hypertension and sympathetic overdrive despite impairment in baroreflex sensitivity. Another key finding relates to the demonstration of the requirement of MRAP2 in MC4R neurons for the control of sympathetic and parasympathetic nervous systems, blood pressure, and heart rate. These findings highlight the critical role of MRAP2 in the regulation of cardiovascular autonomic function.

The body weight phenotype we observed in MC4R^Cre^/MRAP2^fl/fl^ mice is consistent with previous findings showing that loss of MRAP2 globally or in select neuronal populations including MC4R neurons leads to obesity,^18–21^ whereas overexpression of MRAP2 in PVN MC4R neurons results in lower body weight and fat mass in female mice.^22^ The metabolic consequences of MRAP2 deletion extended to glucose homeostasis, with MC4R^Cre^/MRAP2^fl/fl^ mice exhibiting elevated fasting blood glucose levels and impaired glucose tolerance, particularly in males. Both male and female MRAP2-deficient mice showed insulin resistance, as indicated by their diminished response to insulin injections. These results confirm that MRAP2 in MC4R neurons are necessary for energy balance, normal glucose metabolism, and insulin sensitivity, further linking MRAP2 to the regulation of metabolic processes. However, the mechanisms underlying the sex differences noted in some metabolic parameters such as the less marked glucose intolerance in female MC4R^Cre^/MRAP2^fl/fl^ mice is not clear. This may involve sex-specific factors such as oestrogens which have tremendous influence on metabolic functions including glucose metabolism.^34^ Further investigations into the mechanisms driving these sex-specific effects are warranted.

Our investigation into the physiological responses to MC4R activation revealed that MRAP2 deletion alters the anorectic effect of the MC4R agonist, MTII. While MTII reduced food intake and body weight in both control and MRAP2-deficient mice, the effect on food intake at 24 h post-injection was significantly blunted in the MC4R^Cre^/MRAP2^fl/fl^ mice. The lack of difference in the anorectic effect of MTII at 4 h is consistent with the previous observation by Asai *et al.*^19^ that global MRAP2 null mice have unaltered anorectic response to MTII at 90 min. This suggests that MRAP2 specifically modulates certain aspects of MC4R signalling related to appetite suppression. Interestingly, the weight loss response to Celastrol, an agent known to induce significant weight loss in various obesity models, remained effective in the MRAP2-deficient mice.

MRAP2 deletion in MC4R neurons had an impact on SNA. Baseline BAT SNA tended to be lower, and the response to MTII was nearly absent, in both the global (MRAP2^−/−^) and conditional (MC4R^Cre^/MRAP2^fl/fl^) mice. However, it remains unclear why such blunted thermogenic SNA response to MTII did not translate into less MTII-induced weight loss in MC4R^Cre^/MRAP2^fl/fl^ mice. It is possible that developmental aberrations or compensation induced by lifelong loss of MRAP2 in MC4R neurons may have contributed to MTII-induced weight loss in these mice, but this remains to be tested. Consistent with the lower baseline renal SNA, innervation of the kidney was reduced, and the MTII-induced increase in renal SNA was significantly attenuated in MC4R^Cre^/MRAP2^fl/fl^ mice. The reduction in MC4R signalling may explain the lower renal SNA despite the presence of obesity, which typically increases SNA.^11^ Interestingly, MTII-induced increase in hepatic vagal nerve activity was blunted in MC4R^Cre^/MRAP2^fl/fl^ mice. Together, these findings show that MRAP2 deficiency interfere with activity of both the sympathetic and parasympathetic nervous systems. Transsynaptic tracing revealed that MC4R neurons projecting to BAT and kidneys are localized in specific brain nuclei such as the PVN, suggesting that MRAP2 regulates sympathetic outflow through a limited subset of MC4R neurons.

One interesting observation in the current study relates to the absence of hypertension in MC4R^Cre^/MRAP2^fl/fl^ mice despite the presence of obesity. This finding indicates that the hypertension reported previously in patients carrying deficient MRAP2 is probably due to loss of this protein in cell types other than MC4R neurons^20,35–37^ It should be noted that in addition to MC4R, MRAP2 has been implicated in the regulation of trafficking and signalling of number of other G protein-coupled receptors such as the ghrelin receptor.^15,23^ However, the relevance of MRAP2 regulation of these other receptors to blood pressure control and the development of hypertension remains to be determined. Remarkably, despite similar arterial pressure, MRAP2-deficient mice displayed significantly lower heart rate. This decrease in heart rate may be explained by the autonomic imbalance, including an increase in parasympathetic tone (lower LF/HF ratio of heart rate variability), observed in MC4R^Cre^/MRAP2^fl/fl^ mice. The autonomic dysfunction may also explain the attenuated baroreflex sensitivity observed in these mice. For instance, blood pressure is known to be regulated by autonomic nervous system influences on immune organs.^38^ This well-known interactions between the autonomic nervous system and immune system may have also contributed to the other phenotypes we observed in the MC4R^Cre^/MRAP2^fl/fl^ mice.

In conclusion, our results highlight the essential role of MRAP2 in MC4R neurons for maintaining energy balance, glucose homeostasis, and autonomic function. MRAP2 appears to modulate specific aspects of MC4R signalling, particularly those related to appetite suppression and autonomic nervous system activity. Understanding the precise mechanisms by which MRAP2 influences these pathways could offer new therapeutic targets for obesity and metabolic disorders.

Translational perspective Obesity and associated complications particularly hypertension represent major risk factors for cardiovascular disease. This study uncovers the critical role of MRAP2 in MC4R neurons in linking metabolic and cardiovascular regulation. The findings demonstrate that MRAP2 deletion in these neurons reduces sympathetic nerve activity and prevents obesity-associated hypertension. These insights reveal MRAP2 as a key mediator of obesity-induced hypertension and autonomic dysfunction. Targeting MRAP2 pathways may provide novel strategies for treating metabolic and cardiovascular complications of obesity, offering a translational path to address growing global health challenges.

## Supplementary Material

cvaf067_Supplementary_Data

## Data Availability

The data that support the findings of this study are available from the corresponding author upon reasonable request.
