# Supplementary material for: Loss of melanocortin receptor accessory protein 2 in melanocortin-4 receptor neurons protect from obesity-associated autonomic and cardiovascular dysfunctions
Source: Cardiovasc Res. 2025 Apr 17;121(12):1929–40. doi: 10.1093/cvr/cvaf067 (PMC12551390; doi:10.1093/cvr/cvaf067)
Supplement: cvaf067_Supplementary_Data [file cvaf067_supplementary_data.pdf]

Supplemental Material

Loss of MRAP2 in MC4R Neurons Protect from Obesity-Associated Autonomic and Cardiovascular Dysfunctions

Deng Fu Guo[1,2], Paul A. Williams[1], Alexis Olson[1], Donald A Morgan[1,2], Hussein Herz[3], Jon Resch[1,6,7,8], Deniz Atasoy[1,6,7,8], Harald M. Stauss[4], Julien A. Sebag[5,6,7,8], and Kamal Rahmouni[1,2,3,6,7,8*]

1. Department of Neuroscience and Pharmacology, University of Iowa Carver College of Medicine, Iowa City, IA, USA
2. Iowa City Veterans Affairs Health Care System, Iowa City, IA, USA
3. Department of Internal Medicine, University of Iowa Carver College of Medicine, Iowa City, IA, USA
4. Department of Biomedical Sciences, Burrell College of Osteopathic Medicine, Las Cruces, NM, USA
5. Department of Molecular Physiology and Biophysics, University of Iowa Carver College of Medicine, Iowa City, IA, USA
6. Fraternal Order of Eagles Diabetes Research Center, University of Iowa Carver College of Medicine, Iowa City, IA, USA
7. Obesity Research and Educational Initiative, University of Iowa Carver College of Medicine, Iowa City, IA, USA
8. Iowa Neuroscience Institute, University of Iowa Carver College of Medicine, Iowa City, IA, USA

Running title: MC4R Neuron MRAP2

***Corresponding author:**
Kamal Rahmouni, Ph.D.
Department of Neuroscience and Pharmacology
University of Iowa Carver College of Medicine
Iowa City, IA, 52242, USA
e-mail: kamal-rahmouni@uiowa.edu
Tel: 319 353 5256
Fax: 319 353 5350

Supplemental Table 1: Primer sequences used for genotyping.

Number	Name	Forward	Reverse 1
1	Universal Cre	ACCTGAAGATGTTCGCGATTATCT	ACCGTCAGTACGTGAGATATCTT
2	MRAP2	TGCTGAGAAAGTCTGCCCAAGTGT	CTAATGAGTGAATACCTTCTGCT
3	tdTomato-negative	AAGGGAGCTGCAGTGGAGTA	CCGAAAATCTGTGGGAAGTC
4	tdTomato-positive	GGCATTAAAGCAGCGTATCC	CTGTTCCTGTACGGCATGG

Supplemental Table 2: Distribution of tdTomato-labeled MC4R neurons in the brain.

Division	Nucleus/Region	Abbrv	MC4R
Hypothalamus	Anterior hypothalamic area	AH	+
Hypothalamus	Anteroventral periventricular nucleus	AVPe	+
Hypothalamus	Arcuate nucleus	Arc	+
Hypothalamus	Dorsomedial hypothalamic nucleus	DMH	+
Hypothalamus	Interstitial nucleus of the posterior limb of the anterior commissure	IPAC	++
Hypothalamus	Lateral hypothalamus	LH	+
Hypothalamus	Nucleus of lateral olfactory tract	LOT	++
Hypothalamus	Lateral preoptic area	LPO	++
Hypothalamus	Magnocellular preoptic nucleus	MCPO	++
Hypothalamus	Medial tuberal nucleus	MTu	+
Hypothalamus	Median preoptic nucleus	MnPO	+
Hypothalamus	Nucleus of the diagonal band	HDB	+
Hypothalamus	Parasubthalamic nucleus	PSTh	-
Hypothalamus	Paraventricular nucleus	PVN	++
Hypothalamus	Perifornical area	PeF	-
Hypothalamus	Posterior hypothalamic area	PH	+
Hypothalamus	Substantia innominata	SI	-
Hypothalamus	Suprchiasmatic nucleus	SCh	-
Hypothalamus	Ventral pallidum	VP	-
Hypothalamus	Ventral subiculum	VS	++
Hypothalamus	Ventral tuberomammillary nucleus	VTM	-
Hypothalamus	Ventromedial hypothalamic nucleus	VMH	+
Hypothalamus	Ventrolateral preoptic nucleus	VLPO	+
Hypothalamus	Ventromedial preoptic nucleus	VMPO	+
Hypothalamus	Zona Incerta	ZI	-
Brainstem	A5 noradrenergic cell region	A5	+
Brainstem	Caudal ventrolateral medulla	CVLM	+
Brainstem	Cuneate nucleus	Cu	+
Brainstem	Dorsal motor nucleus of vagus	10N	++
Brainstem	Dorsal periolivary region	DPO	-
Brainstem	Dorsal raphe nucleus	DR	-
Brainstem	External cuneate nucleus	ECu	++
Brainstem	Gigantocellular reticular nucleus	Gi	++
Brainstem	Inferior olive	IO	++
Brainstem	Intercalated nucleus	In	-
Brainstem	Intermediate reticular nucleus	IRt	+
Brainstem	Lateral reticular nucleus	LRt	+
Brainstem	Lateral vestibular nucleus	Lve	+
Brainstem	Lobule 1 of the cerebellar vermis	1Cb	+

Brainstem	Locus coeruleus	LC	++
Brainstem	Medial vestibular nucleus	Mve	++
Brainstem	Median Raphe Nucleus	MnR	-
Brainstem	Medioventral periolivary nucleus	MVPO	+
Brainstem	Medullary reticular nucleus, dorsal part	MdD	++
Brainstem	Medullary reticular nucleus, ventral part	MdV	-
Brainstem	Motor trigeminal nucleus	Mo5	-
Brainstem	Nucleus ambiguus	Amb	+
Brainstem	Nucleus of the solitary tract	NTS	+++
Brainstem	Paragiganto cellular nucleus	PGi	-
Brainstem	Paramedian raphe	PMR	-
Brainstem	Paralemniscal nucleus	PL	-
Brainstem	Paratrigeminal nucleus	Pa5	+
Brainstem	Parvicellular reticular nucleus	PCRt	+
Brainstem	Pontine reticular nucleus	PnC	-
Brainstem	Posterodorsal tegemental nucleus	PDTg	+
Brainstem	Prepositus hypoglossal nucleus	Pr	+
Brainstem	Raphe magnus nucleus	RMg	-
Brainstem	Raphe obscurus nucleus	ROb	+
Brainstem	Raphe pallidus nucleus	RPa	+
Brainstem	Rostral periolivary region	RPO	-
Brainstem	Rostral Ventromedial Medulla	RVMM	-
Brainstem	Rostroventrolateral reticular nucleus	RVL	+
Brainstem	Spinal trigeminal nucleus	sp5	+
Brainstem	Subcoeruleus nucleus	SubC	-
Brainstem	Superior paraolivary nucleus	SPO	-
Brainstem	Superior vestibular nucleus	SuVe	-
Brainstem	Rostral ventrolateral medulla	RVLM	-
Brainstem	Ventromedial Medulla	VMM	
Mid-brain	A8 dopamine cells	A8	-
Mid-brain	Aqueduct	Aq	
Mid-brain	Caudal linear nucleus of raphe	CLi	-
Mid-brain	Commissure of inferior colliculus	cic	-
Mid-brain	Cuneiform nucleus	CnF	-
Mid-brain	Deep mesencephalic area (DMN)	DpMe	-
Mid-brain	Edinger-Westphal nucleus	EW	-
Mid-brain	Geniculate nucleus		-
Mid-brain	Inferior colliculus	IC	++
Mid-brain	Interfascicular nucleus	IF	-
Mid-brain	Interpenduncular nucleus	IP	-
Mid-brain	Laterodorsal tegmental nucleus	LDTg	+
Mid-brain	Medial mammillary	MM	+
Mid-brain	Medial terminal nucleus of accessory optic tract	M	-

Mid-brain	Medial pretectal nucleus	MPT	+
Mid-brain	Nucleus of brachium of inferior colliculus	BIC	-
Mid-brain	Nucleus posterior commissure	Pcom	+
Mid-brain	Optic nerve layer of the superior colliculus	Op	-
Mid-brain	Parabrachial nucleus	PB	-
Mid-brain	Parvocellular oculomotor nucleus	P	-
Mid-brain	Premammillary nucleus	PM	+
Mid-brain	Pedunculopontine tegmental nucleus	PPTg	-
Mid-brain	Periaqueductal gray	PAG	+
Mid-brain	Precommisural nucleus	PrC	+
Mid-brain	Premammillary nucleus, ventral	PMV	-
Mid-brain	Red necleus	R	-
Mid-brain	Retromammillary nucleus	RM	+
Mid-brain	Retrorubral field	RRF	-
Mid-brain	Rostroal linear nucleurs of the raphe	RLi	-
Mid-brain	Substantia nigra	SN	-
Mid-brain	Superior colliculus	SC	++
Mid-brain	Supraoculomotor cap	Su3C	-
Mid-brain	Supraoculomotor central gray	Su3	-
Mid-brain	Tegmental nucleus	Tg	+
Mid-brain	Ventral tegmental area	VTA	-
Cortex	Auditory cortex	Au	++
Cortex	Agranular Insular Cortex	AI	+++
Cortex	Anterior olfactory area, lateral part	AO	++
Cortex	Cingulate cortex	Cg1	++
Cortex	Claustrum	CI	++
Cortex	Dorsal endopiriform nucleus	DEn	++
Cortex	Dentate Gyrus	DG	++
Cortex	Dorsolateral entorhinal cortex	DLEnt	++
Cortex	Ectorrhinal cortex	Ect	++
Cortex	Fields CA1, CA2, & CA3 of the Hippocampus	CA1	++
Cortex	Frontal association cortex	FrA	++
Cortex	Granular insular cortex	GI	+++
Cortex	Granule cell layer of the olfactory bulb	GrO	++
Cortex	Infralimbic cortex	IL	+
Cortex	Insular cortex	Insular	+++
Cortex	island of Calleja?	ICj	++
Cortex	Lateral entorhinal cortex	LEnt	-
Cortex	Olfactory tubercle	Tu	++
Cortex	Orbital cortex	O	+++
Cortex	Perirhinal cortex	PRh	++
Cortex	Piriform cortex	Pir	++
Cortex	Prefrontal cortex	PFC	-

Cortex	Prelimbic cortex	PrL	++
Cortex	Primary motor cortex	M1	+++
Cortex	Pyramidal cell layer of the hippocampus	Py	++
Cortex	Secondary motor (Premotor) cortex	M2	+++
Cortex	Somatosensory	S1	+++
Cortex	Temporal cortex, Association area	TeA	++
Cortex	Ventral endopiriform nucleus	VEn	++
Cortex	Visual cortex	V1	++
Circumventricular	Area postrema	AP	+++
Circumventricular	Median eminence	ME	-
Circumventricular	Organum vasculosum lamina terminalis	OVLT	-
Circumventricular	Subfornical Organ	SFO	+
Thalamus	Anteromedial thalamic nucleus	AM	-
Thalamus	Central medial thalamic nucleus	CM	-
Thalamus	Lateral habenula	LHb	-
Thalamus	Paraventricular thalamic nucleus	PVT	-
Thalamus	Reticular thalamic nucleus	Rt	-
Thalamus	Reuniens thalamic nucleus	Re	-
Thalamus	Submedius thalamic nucleus	Sub	-
Thalamus	Subparafascicular thalamic nucleus	SPF	-
Thalamus	Ventral anterior thalamic nucleus	VA	-
Thalamus	Ventral geniculate nucleus	VLG	-
Substriatum	Bed nucleus of the stria teminalis	BNST	++
Substriatum	Nucleus accumbens	Acb	-
Substriatum	Parastrial nucleus	PS	-
Amygdala	Amygdala, central part	AMY	
Amygdala	Amygdalohippocampal transition area	Ahi	++
Amygdala	Amygdalopiriform transition area	APir	++
Amygdala	Anterior amygdaloid area	AA	+
Amygdala	Anterior cortical amygdaloid area	ACo	++
Amygdala	Basomedial amygdaloid nucleus	BMA	++
Amygdala	Basolateral amygdaloid	BLA	++
Amygdala	Central amygdaloid nucleus	CeC	+
Amygdala	Lateral nucelus of the amydala	LA	+
Amygdala	Medial amygdaloid nucleus	MeA	+
Amygdala	Posterolateral cortical nucleus of the amygdala	PLCo	+++
Septal area	Dorsal penducular cortex	DP	++
Septal area	Lateral septal nucleus	LSD	++
Septal area	Medial septal nucleus	MS	+
Septal area	Septofimbrial nucleus	SFi	++
Septal area	Septohippocampal nucleus	SHi	+

Supplemental Figures

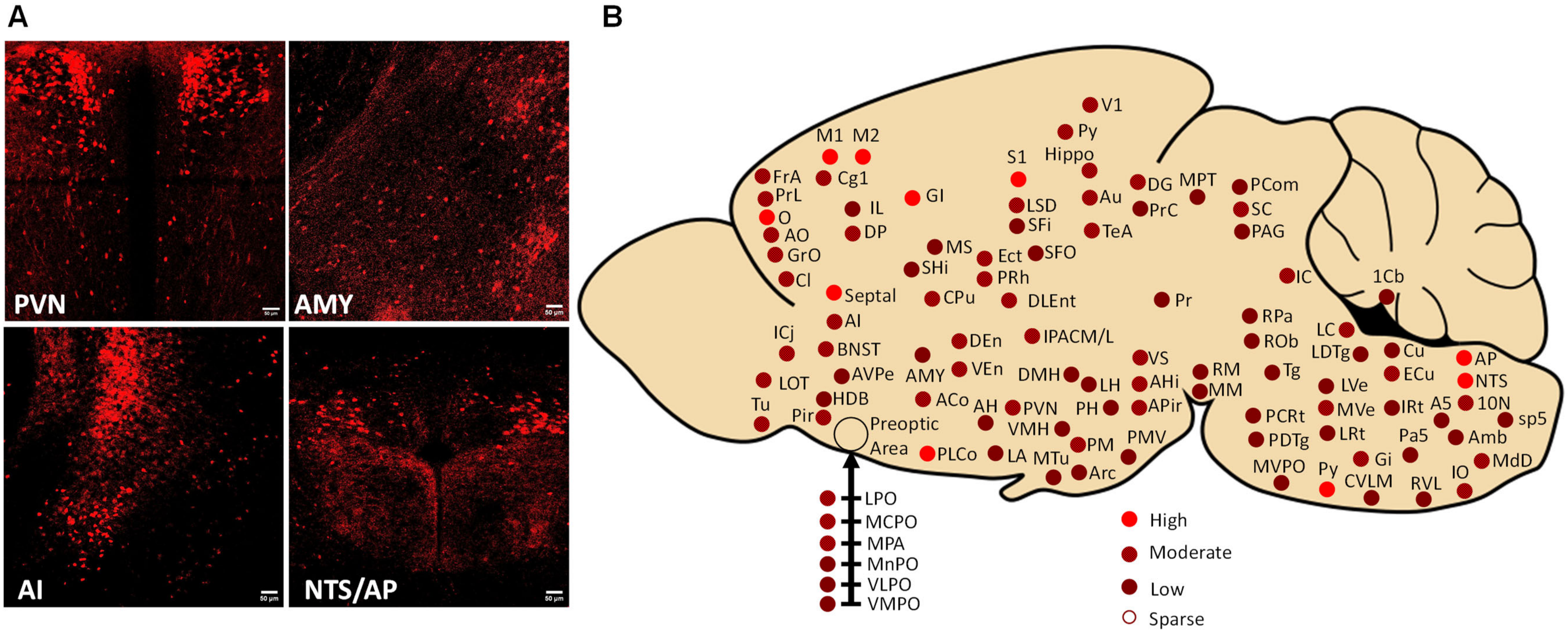

Supplemental Figure 1: Distribution of MC4R-expressing neurons in the brain. **A**) Representative images showing tdTomato-labeled MC4R neurons in the paraventricular nucleus of the hypothalamus (PVN), nucleus tractus solitarius (NTS)/area postrema (AP), agranular insular cortex (AI), and amygdala (AMY). (B) Diagram depicting the nuclei where tdTomato-labeled MC4R are detected throughout the brain including the cortex, hippocampus, thalamus, hypothalamus, midbrain, and brainstem regions (see Supplemental Table 2 for the abbreviations of the various nuclei). Scale bars: 50 μm.

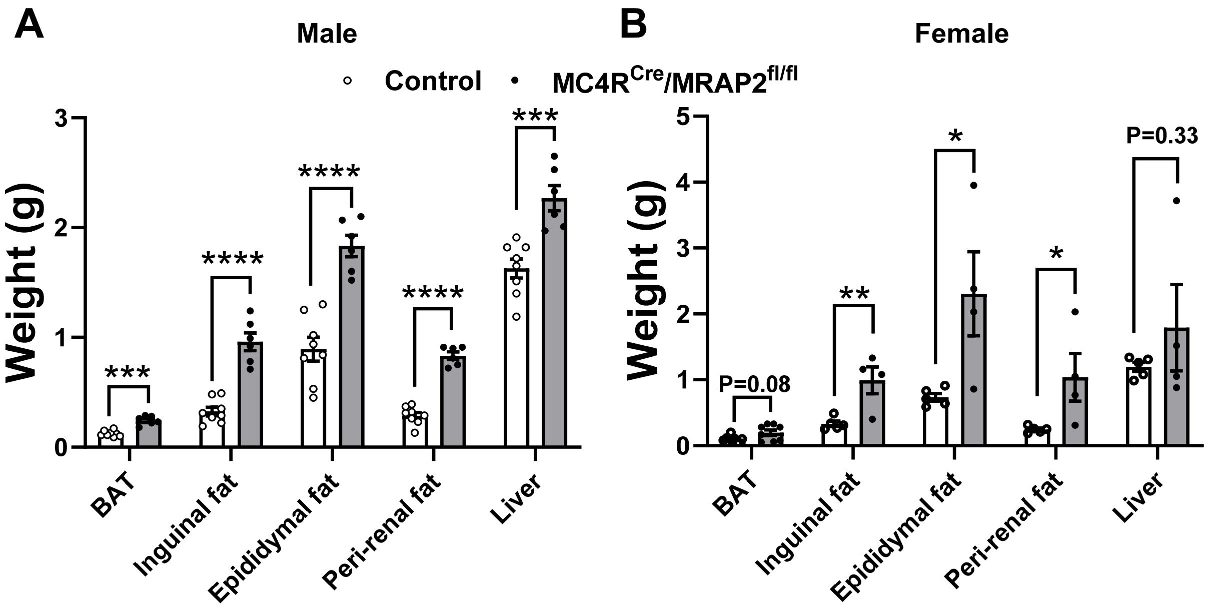

Supplemental Figure 2: Loss of MRAP2 in MC4R neurons causes obesity. (A–B) Weights of different fat pads and liver of male (A) and female (B) mice. Student's *t*-test was used for statistical analysis. *$P < 0.05$, **$P < 0.01$, ***$P < 0.001$ and ****$P < 0.0001$.

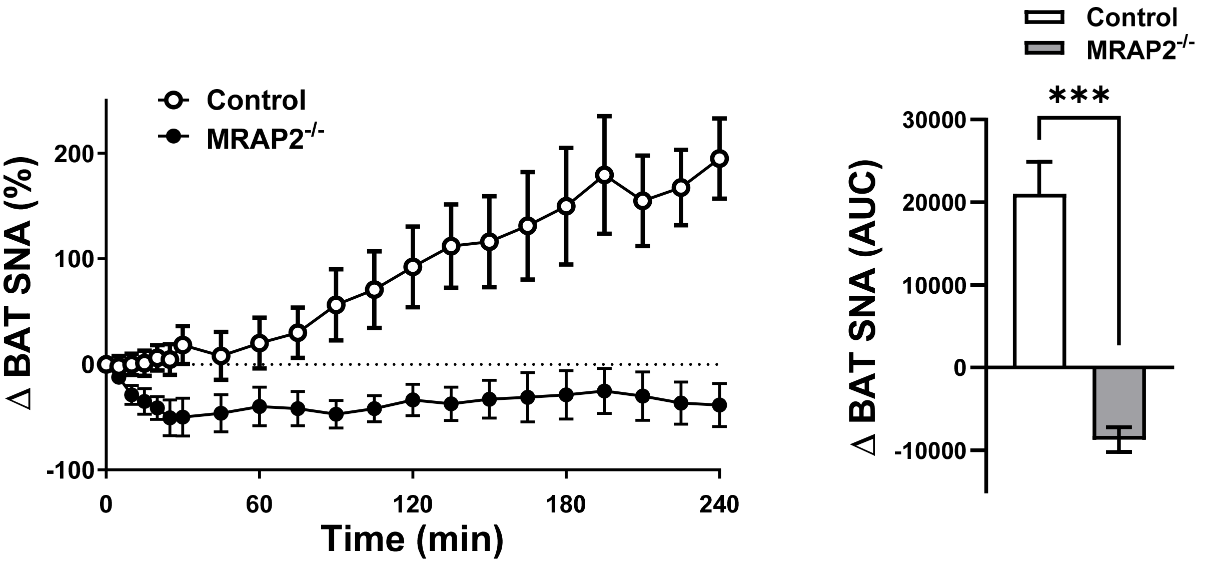

Supplemental Figure 3: Global MRAP2 deficiency attenuates the thermogenic sympathetic responses to MC4R stimulation. Effect of MTII (ICV, 2 µg) vs vehicle on BAT SNA of control (n = 6) and MRAP2$^{-/-}$ (n = 4) male mice. Two-way ANOVA with repeated measure or Student's *t*-test was used for statistical analysis. ****P* < 0.001.

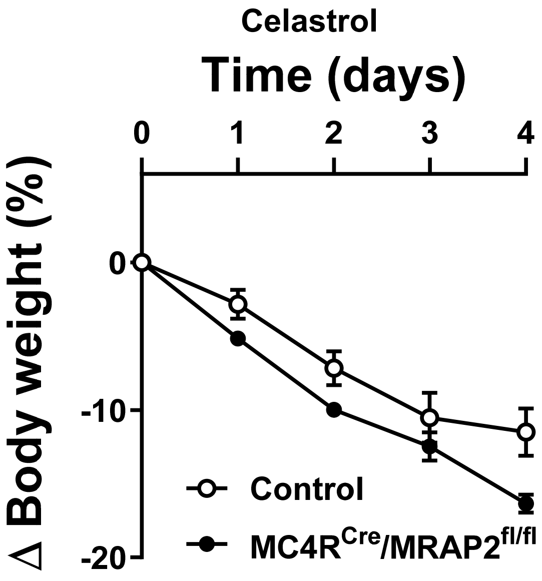

Supplemental Figure 4. Comparison of the percent change in body weight between control (n = 6) and MRAP2$^{-/-}$ (n = 4) male mice treated with Celastrol (IP, 0.5 mg/kg body weight, twice daily for 4 days). Two-way ANOVA with repeated measure was used for statistical analysis.

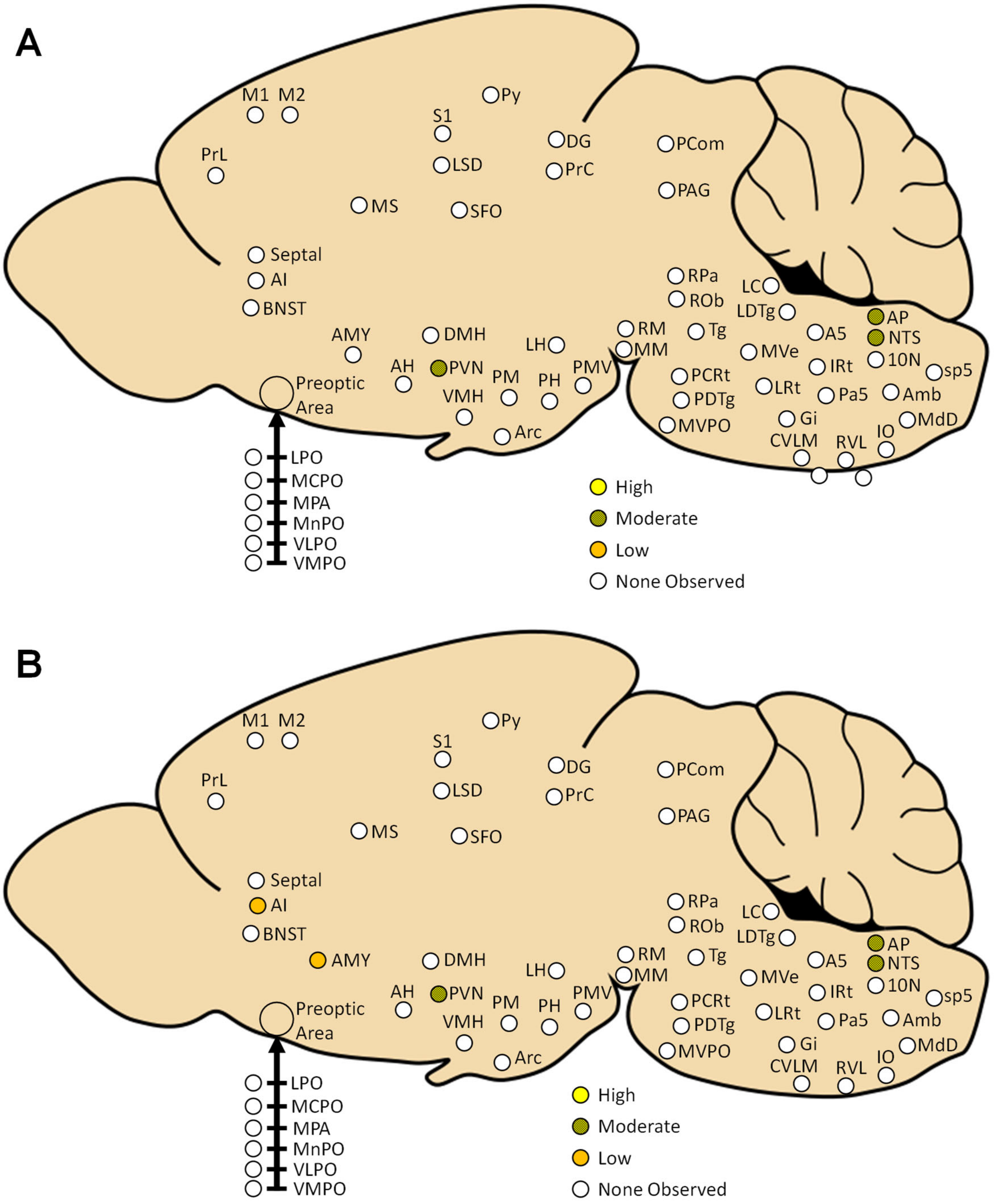

Supplemental Figure 5. (B) Diagrams summarizing brain nuclei containing MC4R neurons that project to BAT (A, n = 6 (3 males, 3 females)) or kidneys (B, n = 7 (4 males, 3 females)). Note that the nuclei that contained MC4R neurons but did not exhibit PRV-GFP staining (none observed) are also shown as white dots.

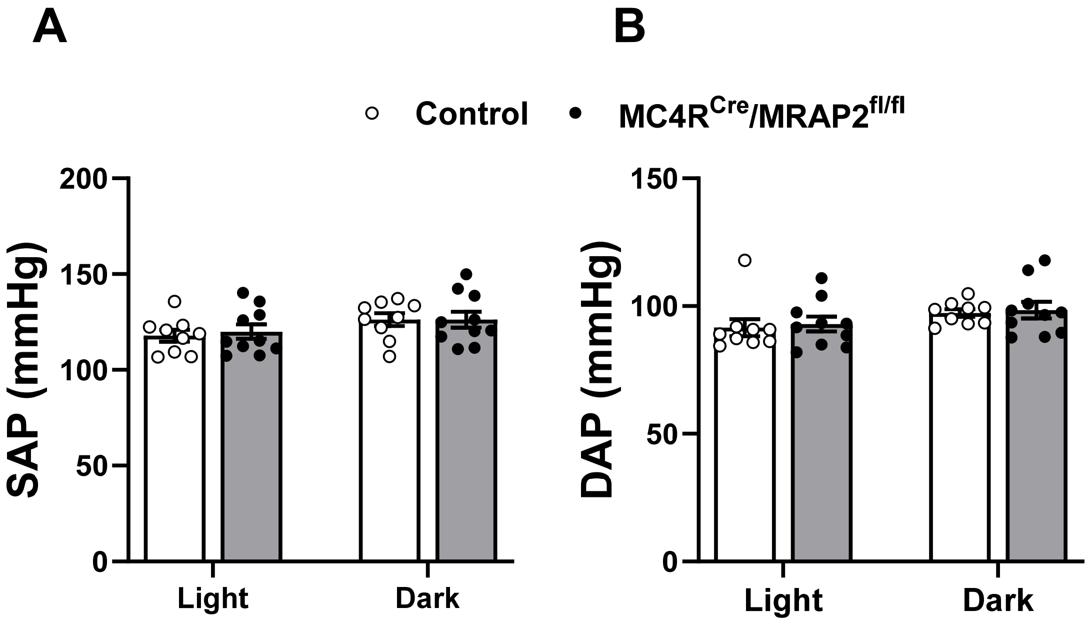

Supplemental Figure 6. MC4R neuron MRAP2 deficiency protects from hypertension. (A–B) Baseline telemetry systolic (SAP, A) and diastolic (DAP, B) arterial pressure of control and $MC4R^{Cre}/MRAP2^{fl/fl}$ male mice. Student's *t*-test was used for statistical analysis.

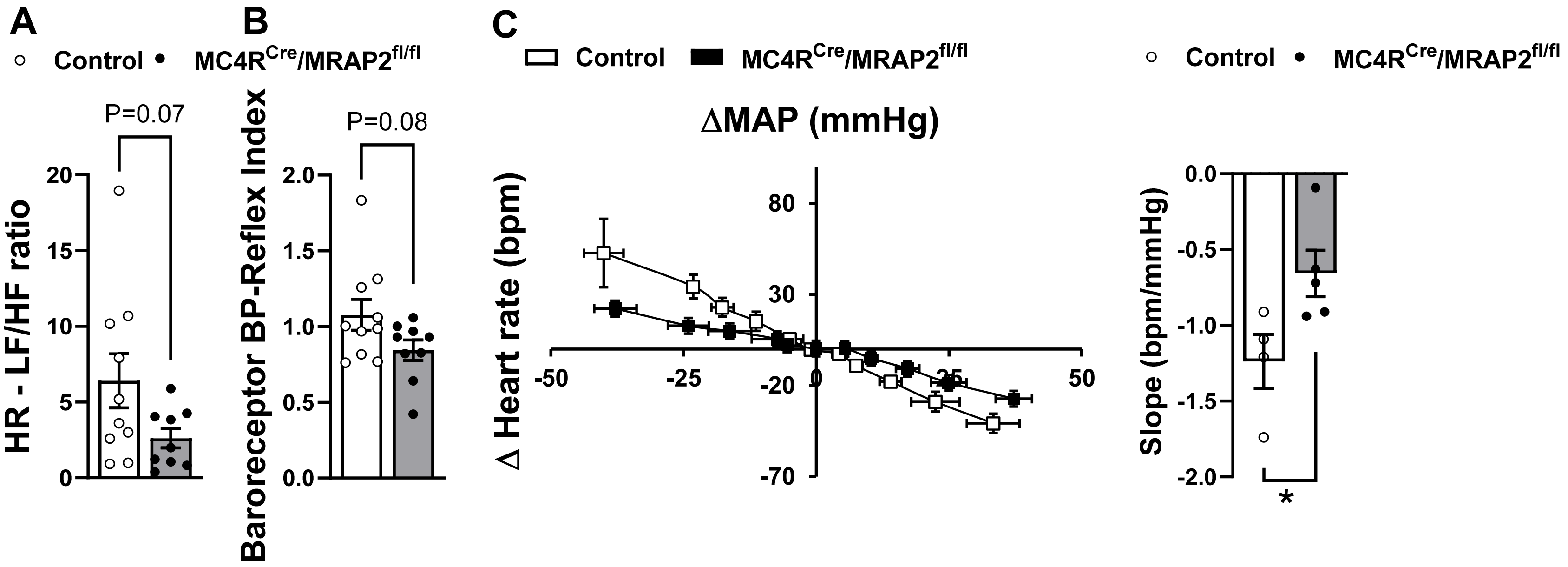

Supplemental Figure 7. (A-B) Power spectral analysis of heart rate (HR) variability (ratio of low- to high-frequency (LF/HF) components) (A) and baroreflex blood pressure (BP) reflex index (B) of control and MC4R^{Cre}/MRAP2$^{fl/fl}$ male mice. (C) Baroreceptor reflex sensitivity based on the change in heart rate evoked by the increase and decrease in mean arterial pressure (MAP) induced by phenylephrine and sodium nitroprusside, respectively, and related slope of control and MC4R^{Cre}/MRAP2$^{fl/fl}$ male mice. Two-way ANOVA with repeated measure or Student's *t*-test was used for statistical analysis. *$P < 0.05$.
